# High pathogenicity of emerging porcine G9P[23] and G11P[7] rotavirus for newborn piglets in China

**DOI:** 10.3389/fvets.2025.1531861

**Published:** 2025-04-02

**Authors:** Zhendong Zhang, Duo Li, Sun He, Jiubin Du, Yubo Li, Qiangde Liu, Pengjiang Wang, Wenqiang Wang, Wei Wen, Zhenbang Zhu, Xudong Tang, Xiangdong Li

**Affiliations:** ^1^Jiangsu Co-Innovation Center for Prevention and Control of Important Animal Infectious Diseases and Zoonoses, College of Veterinary Medicine, Yangzhou University, Yangzhou, China; ^2^School of Biotechnology, Jiangsu University of Science and Technology, Zhenjiang, China; ^3^TECON Biopharmaceutical Co., Ltd., Ürümqi, China

**Keywords:** porcine group A rotavirus, G9P[23], G11P[7], pathogenicity, piglets

## Abstract

In order to better understand the pathogenicity of the current porcine A group rotavirus (PoRVA) field strains, AHBZ2304 (G9P[23]) and AHBZ2303 (G11P[7]) isolated from diarrhea suckling piglets were selected for pathogenicity analysis in the present study. Experimental inoculation of colostrum-deprived 2-day-old piglets revealed that both isolates caused severe clinical sings, high level of virus shedding and significant damage to the small intestinal villi. Additionally, both gross and microscopic lung lesions were identified at 72 h post infection (HPI) compared to control. Alterations in the microbiota and the overexpression of inflammatory cytokines may serve as critical mechanisms driving the bowel disease associated with PoRVA infection. Our results are of great significance for understanding the pathogenicity of PoRVA emerged in recent years, highlighting the potential for porcine rotavirus to become epidemic and complex, and necessitating heightened attention of the often-overlooked disease in the field.

## 1 Introduction

Porcine rotavirus (PoRV) was first isolated from the infected pigs in 1976, primarily targeting and disrupting mature enterocytes and enteroendocrine cells in the small intestine, which results in watery diarrhea, weight loss, and dehydration for suckling piglets (1, 2). PoRV exhibits rapid evolution and high genetic diversity within the global swine population. In recent years, there has been a notable increase in the detection rate, prevalence, and incidence of PoRV, posing new challenges for the swine industry in China (3, 4). The G and P genotype classification, based on the sequence identities of VP7 and VP4, has been widely adopted to monitor the prevalence and evolution of PoRV (5). In China, the G5 genotype has been the most prevalent. However, several novel genotypes have emerged, indicating a potential shift in dominance within pig herds (4, 6, 7). Qiao et al. (4) performed a nationwide epidemiological investigation of PoRV in 2022, and the results showed that G9P[23] was the most prevalent genotype combination in China. Despite this, studies on the pathogenicity of G9P[23] has remained largely unreported. G11 rotaviruses were reported to be of porcine origin and the first G11 rotavirus strain YM was isolated in 1983 from piglets in Mexico (8, 9). Most human G11 rotaviruses have been identified from reassortment events between porcine rotaviruses and human Wa-like strain (10). The prevalence and positive rate of porcine G11 rotaviruses have been relatively low, resulting in limited attention and few reports of G11 strains in China. One G9P[23] PoRV strain AHBZ2304 and one G11P[7] PoRV strain AHBZ2303 were isolated from diarrhea piglets in our previous study (11), and the pathogenicity was investigated using 2-day-old piglets in the present study.

## 2 Materials and methods

The PoRVA strain AHBZ2303 and AHBZ2304 propagated in MA104 cells were inoculated onto a 48-well plate at a multiplicity of infection (MOI) of 0.1, and the cells and supernatants were collected at 12, 24, 36, 48, 60, and 72 HPI. Growth kinetics were performed in MA104 cells measured by 10-fold serial dilution at 48 HPI by indirect immunofluorescence assay (IFA) and the 50% tissue culture infectious dose (TCID_50_) per mL was calculated using the Reed–Muench method as described before (12). Besides, cells were harvested and lysed in a cell lysis buffer (Beyotime, China) for western blot analysis of VP6 protein using polyclonal antibody (1:2,000) prepared in our lab.

The animal experiment was approved by the animal welfare and ethics committee of TECON Biology Co., Ltd. (TBCA20-014). All animals were handled following the Experimental Animal Regulation Ordinances defined by the Chinese National Science and Technology Commission. Humane care and healthful conditions were provided for the animals. Thirteen colostrum-deprived piglets confirmed negative for PoRV, PEDV, TGEV, and PDCoV by RT-PCR in the absence of diarrhea were randomized into three groups. All the piglets were raised in separate rooms and fed with milk power every 3 h throughout the experiment artificially at 33°C in an incubator. Among them, three were in the negative control group, five in AHBZ2303 infection group and five in AHBZ2304 infection group. The piglets were orally inoculated with the virus (4 mL/piglets, 10^7^ TCID_50_/mL) or DMEM (4 mL/piglets) after 1 day adaptive cultivation. All piglets were monitored for clinical signs and diarrhea occurrence every 12 h and the severity was scored. The evaluation standards of diarrhea score: 0, normal; 1, soft (cowpie); 2, very soft or light liquid; 3, liquid with some solid content; 4, watery diarrhea with no solid content. The criteria of clinical mental state score: 0, normal; 1, mild lethargy (slow move; head down); 2, moderate lethargy (stands but tends to lie down); 3, heavier lethargy (lies down; occasionally stands) (13). Fecal swabs were collected in 0.5 mL PBS every 12 h and tissue samples were collected at euthanasia using pentobarbital sodium (150 mg/kg), which were extracted to evaluate virus load using SYBR Green qRT-PCR targeting nsp5 gene established in our lab. The fixed lung, jejunum, ileum and colon were acquired to perform histopathological and immunohistochemical examinations targeting PoRV-specific antigen using VP6 polyclonal antibody prepared in our lab. Besides, jejunal and colonic content were collected to perform 16S rRNA gene sequencing for microbiome analysis at Genedenovo Biotechnology Co., Ltd. (Guangzhou, China), and the production of inflammatory cytokines in the jejunum were detected using qRT-PCR. The primers used in the study was listed in Supplementary Table S1. The results were expressed as the mean ± standard deviation (SD) of three independent experiments.

## 3 Results

### 3.1 Virus titration and growth characterization

**Figure 1:**
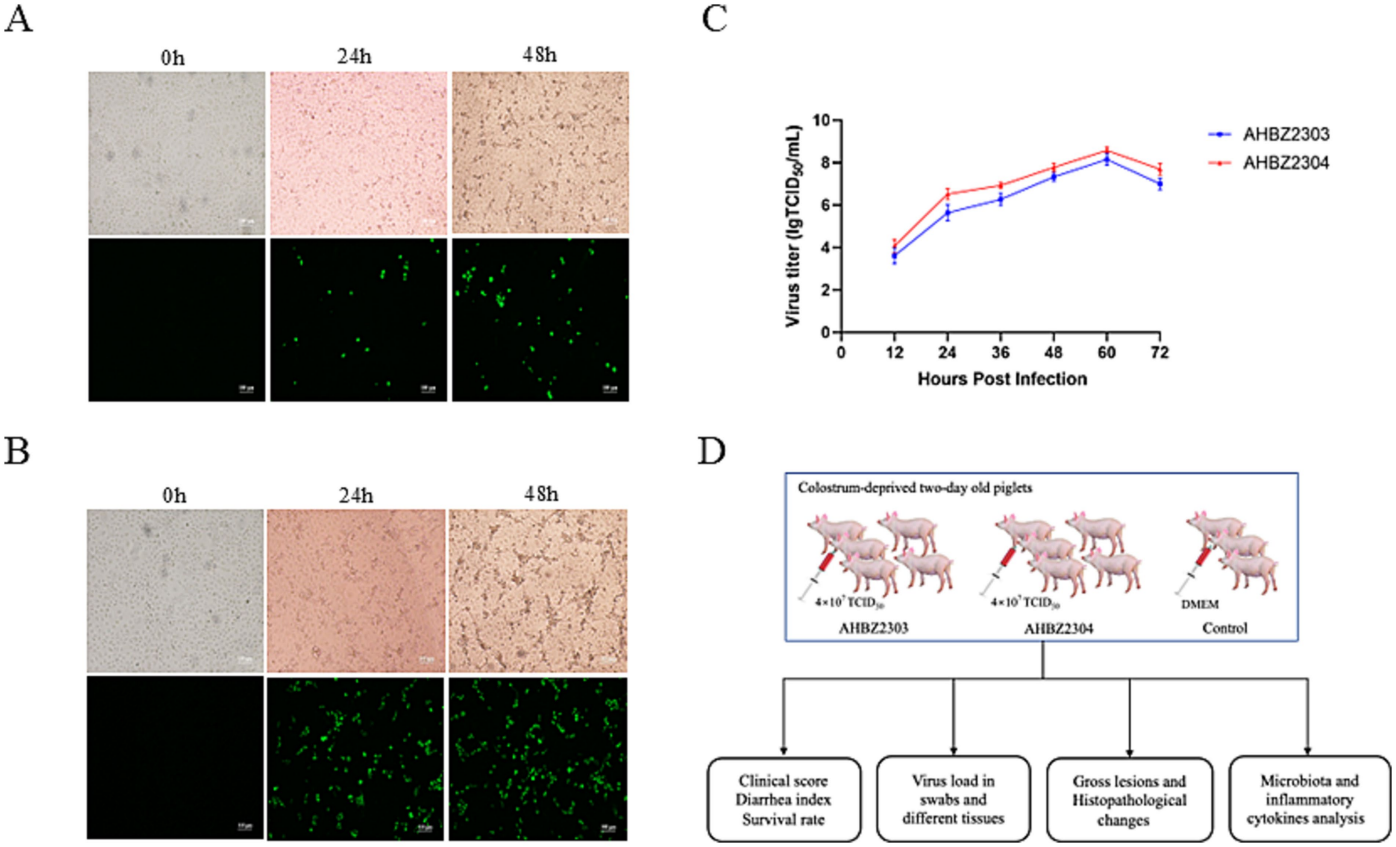
Biological characterization of AHBZ2303 and AHBZ2304 strains in MA104 cells. CPEs and immunostaining analysis of VP6 protein expression in MA104 cells infected with AHBZ2303 **(A)** and AHBZ2304 **(B)**. Viral growth kinetics of AHBZ2303 and AHBZ2304 at a multiplicity of infection (MOI) = 0.1 in MA104 cells. Cells and supernatants were harvested at 12, 24, 36, 48, 60, and 72 HPI and titrated with 50% tissue culture infectious dose assays **(C)**. Data was presented as mean ± SD by triplicates. The overall plan for the animal experiment **(D)**.

AHBZ2303 and AHBZ2304, isolated from MA104 cells, were adapted to the cell line through serial passage. Cytopathic effects (CPE) and positive staining signals for the VP6 protein as demonstrated by immunofluorescence assay (IFA) were obviously observed at 24 and 48 h post infection as shown in Figures 1A,B. The virus of passage 6 was stocked and growth kinetics was analyzed, both two isolates exhibited efficient replication ability, with maximum virus titers reaching 10^8.16^ TCID_50_ and 10^8.58^ TCID_50_ per mL at 60 h post infection (HPI) (Figure 1C). Furthermore, the structural protein VP6 can also be observed through western blot (Supplementary Figure S1). Overall plan for the animal experiment is shown in Figure 1D.

### 3.2 Clinical signs, virus RNA in the fecal swabs and tissues, and inflammatory cytokines analysis

**Figure 2:**
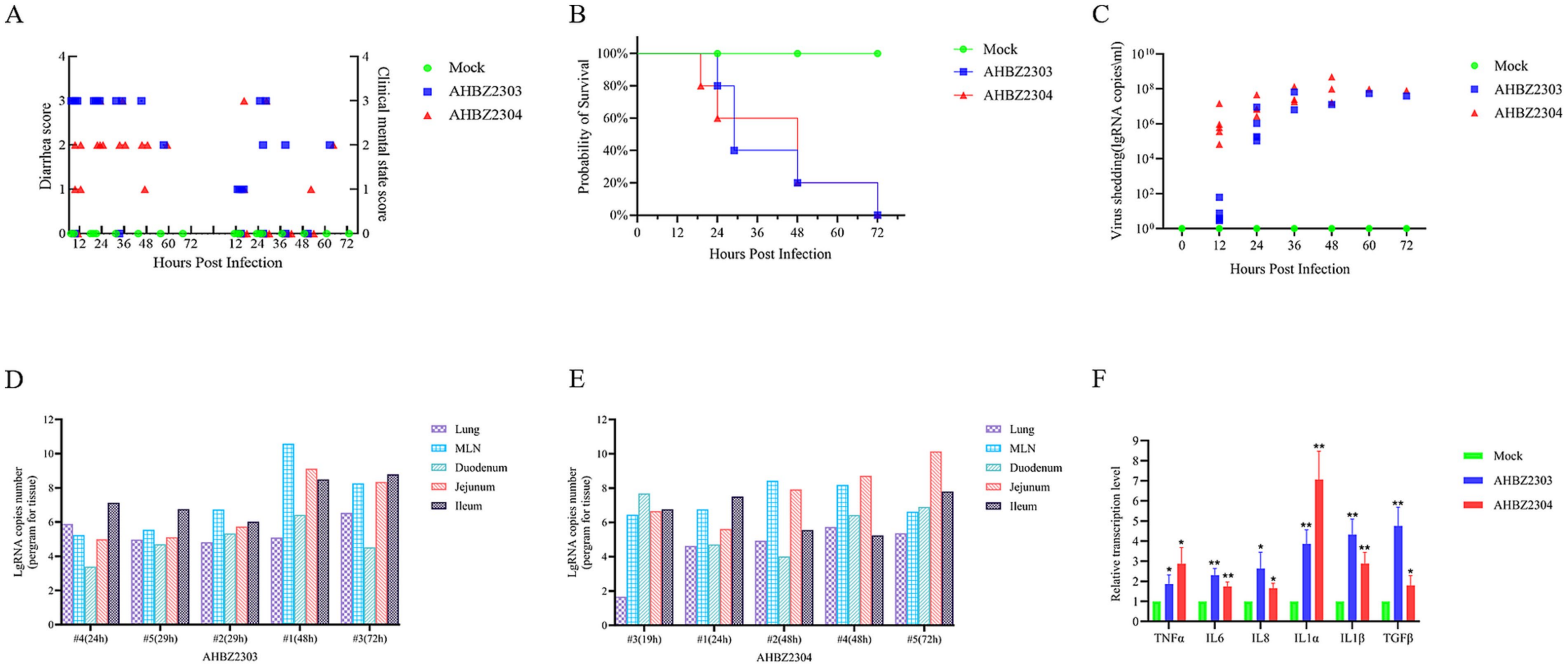
Clinical symptoms and viral load of piglets infected with AHBZ2303 and AHBZ2304. **(A)** Diarrhea index and clinical score were recorded every 12 h. **(B)** Survival rate curve of experimental piglets. **(C)** Quantification of fecal viral shedding. The fecal swabs were acquired every 12 h and the viral load was assessed by qPCR. **(D,E)** Qualification of viral abundance (RNA copy number per mg) in lungs, MLNs, duodenum, jejunum and ileum using qPCR after necropsy. “#4 (24 h)” means the numbered 4 piglet died in 24 HPI. **(F)** The analysis of inflammatory cytokines. Qualification of inflammatory cytokines in jejunum using qPCR compared to control piglets at 24 HPI. The $2^{-\mathrm{\Delta \Delta CT}}$ method was used to calculate the relative mRNA level of target genes.

The piglets of mock-infected group did not develop any symptoms of diarrhea and kept healthy during the experiment, but 4/5 challenged piglets in AHBZ2303 and AHBZ2304 group showed mild to severe diarrhea manifested as loose or watery stool at 12 HPI, and all challenged piglets occurred diarrhea at 24 HPI (Figure 2A). One piglet of AHBZ2304 group succumbed at 19 HPI and one piglet of AHBZ2303 group died at 24 HPI. All the rest of infected piglets died within 72 HPI (Figure 2B). Virus shedding in the fecal swabs and virus distribution in lung, mesenteric lymph nodes (MLNs), duodenum, jejunum and ileum were determined using qRT-PCR targeting nsp5 gene. qRT-PCR results showed that viral RNA could be detectable in the swabs as early as 12 HPI, and continued to increase with the peak of 5.45 × 10^7^ copies/mL (*n* = 1) for AHBZ2303 at 60 HPI and 2.01 × 10^8^ copies/mL (*n* = 3) for AHBZ2304 at 48 HPI (Figure 2C). As shown in Figures 2D,E, the viral RNA could be detected in all the tested tissues from the infected piglets died at different time, and high RNA copies were observed in jejunums, ileums and MLNs. No viral RNA was detected in the swabs and tissues of piglets in the control group. The levels of inflammatory cytokines, including interleukin (IL)-6, IL-8, IL-1α, IL-1β, TGF-β and tumor necrosis factor (TNF)-α were assessed in the jejunum using the qRT-PCR. The results indicated significant expression of these cytokines in AHBZ2303 or AHBZ2304 infected piglets than the piglets in control group (Figure 2F).

### 3.3 Gross lesions and histopathological changes

**Figure 3:**
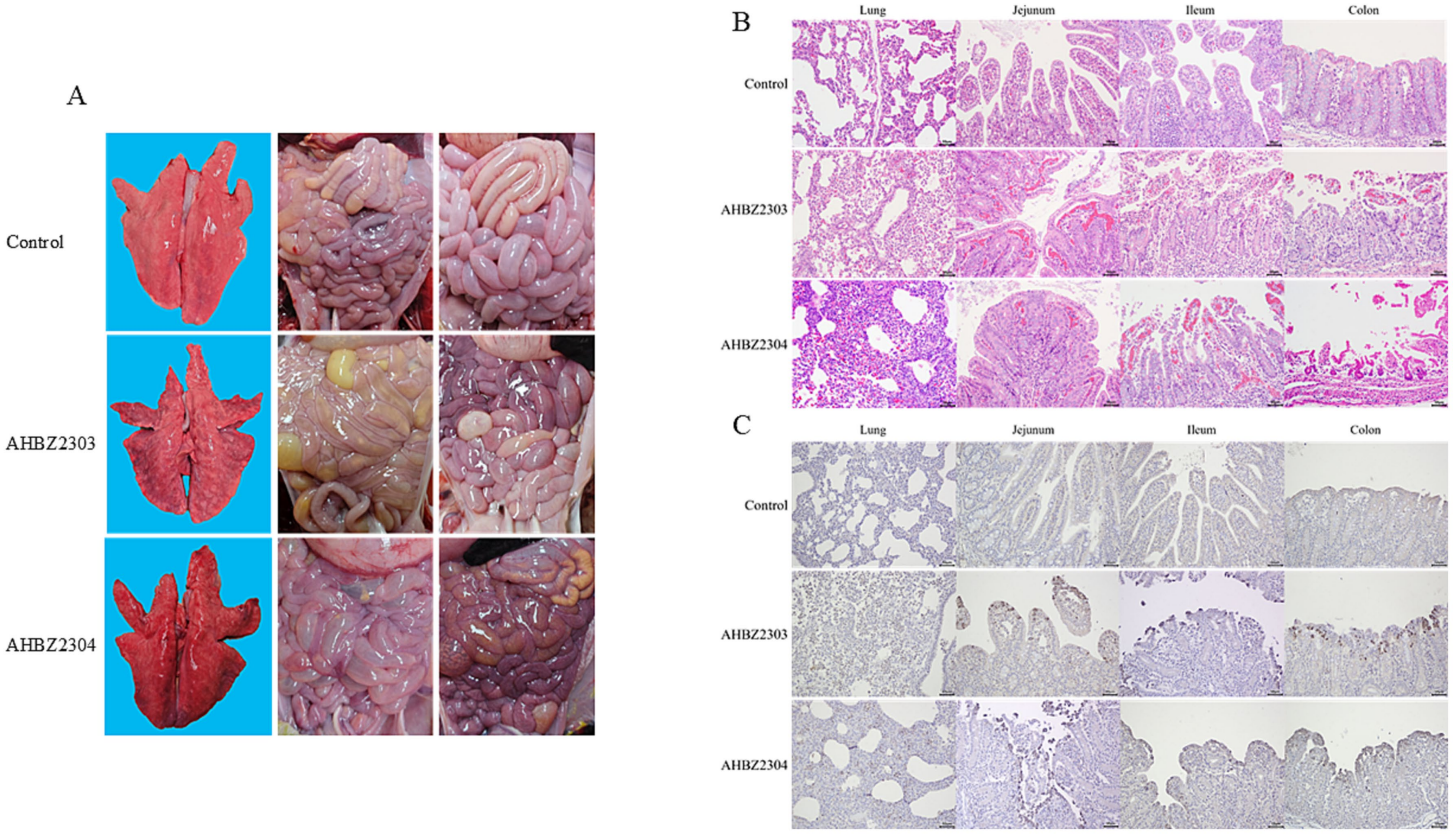
The representative images of macroscopic lesions **(A)**, histopathological lesions **(B)** and immunohistochemical staining **(C)** of intestinal and lung sections infected with AHBZ2303 or AHBZ2304.

Compared to the control group, gross lesions were observed in the groups infected with AHBZ2303or AHBZ2304 during autopsy, characterized by transparent and thin intestinal wall, yellow water-like and hemorrhagic liquid, and inflated intestine (Figure 3A). Notably, the lungs of infected piglets that died at 72 HPI showed macroscopical lesions with pulmonary consolidation and congestion (Figure 3A), while no obvious pathological changes were found in other extra-intestinal tissues. As shown in Figure 3B, histologic observations revealed that the alveolar interstitial thickening and inflammatory cell infiltration, suggesting interstitial pneumonia resulting from AHBZ2303 or AHBZ2304 infection. Additionally, pronounced microscopic intestinal lesions were also identified, including hyperaemia in lamina propria, villous atrophy and fusion, and villous epithelial desquamation (Figure 3B). Immunohistochemical examinations further demonstrated that specific VP6 antigen was dominantly localized in the epithelial cells of the atrophied villi, also the positive signal was identified in the infected lungs (Figure 3C).

### 3.4 Microbiota composition

**Figure 4:**
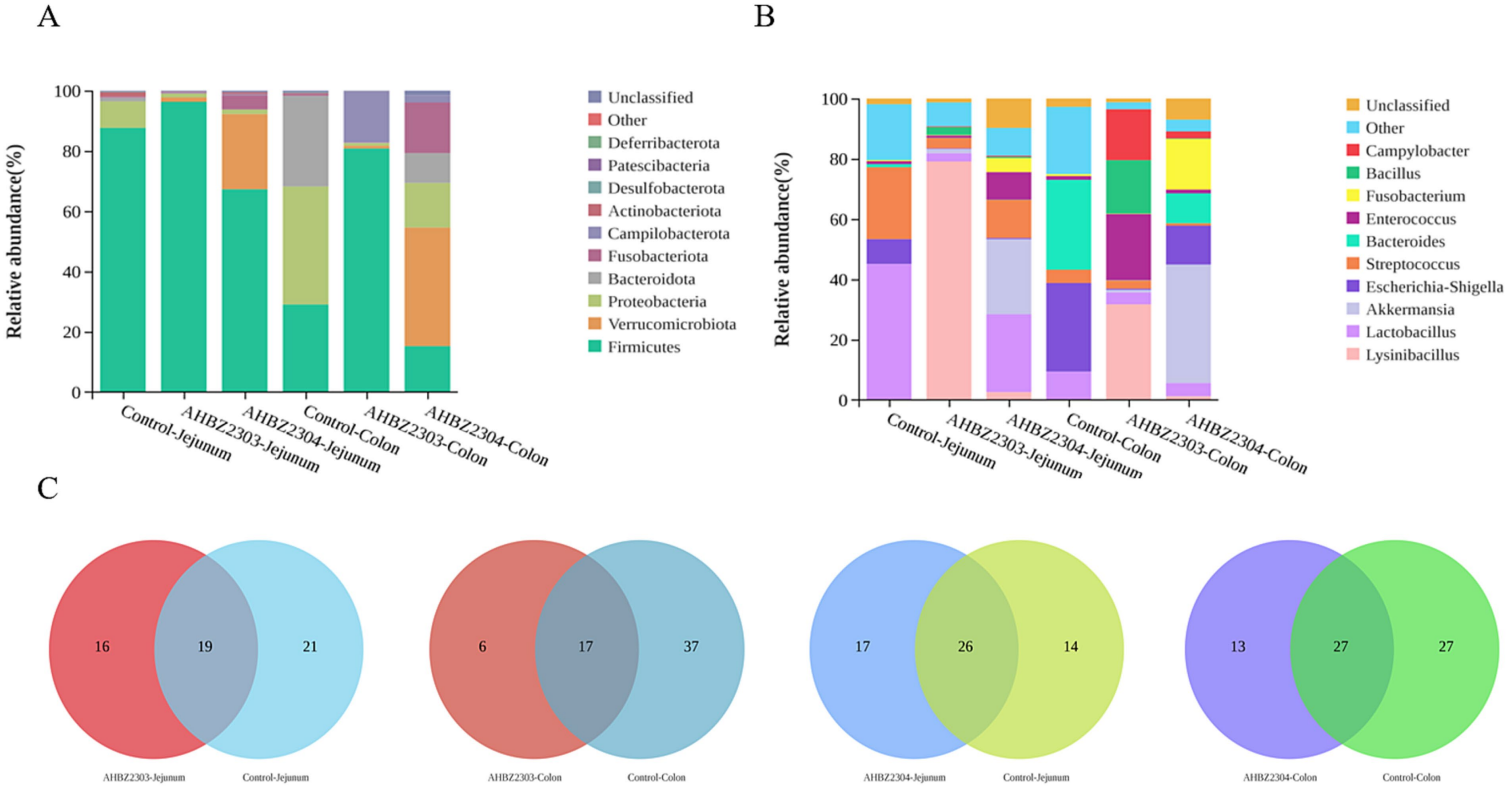
The analysis of microbiota changes. The differences in the abundance of bacterial at the phylum **(A)** and genus **(B)** levels were analyzed. The Venn diagram showed the shared and unique bacterial genera in the control and virus infected jejunum and ileum **(C)**.

A total of 12 fecal samples were collected from the jejunum and colon of both control and died piglets at 24 HPI for 16S rRNA sequencing and microbiota analysis. As shown in Figure 4A, at the phylum level, *Firmicutes* was the most predominant phylum in jejunum across all group; however, the abundance of *Verrucomicrobiota* and *Fusobacteria* was significantly increased in the jejunum of piglets infected with AHBZ2304. In the colon, compared to the control group, piglets infected with AHBZ2303 exhibited elevated levels of *Campilobacterota* and *Firmicutes* levels, while those infected with AHBZ2304 showed higher levels of *Verrucomicrobiota* and *Fusobacteriota*, alongside significantly lower levels of *Firmicutes*, *Proteobacteria*, and *Bacteroidota*. At the genus level (Figure 4B), *Lactobacillus* and *Streptococcus* were reduced, whereas *Lysinibacillus* and *Akkermansia* showed significant increases in the jejunum of piglets infected with AHBZ2303 or AHBZ2304, respectively. Similarly, large difference was determined about the composition in colon between control and virus-infected groups. Venn diagram analysis revealed AHBZ2303 had 16 and 6 unique genera in jejunum and colon, while AHBZ2304 had 17 and 13 unique genera in jejunum and colon, respectively (Figure 4C).

## 4 Discussion

Tian et al. (14) conducted a systematic review identifying 92 rotavirus outbreaks and 96,128 human cases in China from 1982 to 2021. As a zoonotic disease, no much attention was taken to investigate the epidemiological characteristics and outbreaks of porcine rotavirus (PoRV) before 2020 in China. Recently, however, PoRV has re-emerged in Chinese pig herds, exhibiting morbidity rates ranging from 10 to 60%, despite this, research on the pathogenicity of these newly emerged strains remains scarce. Kim et al. (15) firstly performed the pathogenicity of PRG942 (G9P[23]) and PRG9121 (G9P[7]) in a pig mode and found they were highly virulent for piglets. Wang et al. (16) reported severe watery diarrhea in 3-day-old piglets infected with HN03 (G9P[23]) within 24 h and recovery after 72 h. Similarly, the AHFY2022 (G9P[23]) strain caused severe diarrhea and intestinal damage in 5-day-old and 27-day-old piglets, with no died piglets during the experiments (12). In contrast, Gao et al. (17) found that infection of 15-day-old piglets with the JS strain (G5P[23]) resulted in a mortality rate of 37.5% (3 of 8). Additionally, a previous study found emerged G12P[7] strain CN127 infected piglets became lethargic from 60 HPI to 86 HPI, while another study indicated a mortality rate of 66.67% in 1-day-old piglets infected with the G9P[23] strain GX9579 by 120 h (18). The pathogenicity of various PoRV strains for piglets isolated in China is summarized in Table 1. In this study, we analyzed the pathogenicity of AHBZ2303 (G11P[7]) and AHBZ2304 (G9P[23]) isolated from our previous study, which exhibited high virulence, resulting in 100% mortality in newborn colostrum-deprived piglets. As shown in Table 1, combing the results of AHBZ2304, the emerged predominant G9P[23] exhibited variations in pathogenicity. Performing concurrent animal experiments using different G9P[23] strains and analyzing the pathogenic genes contributing the difference in the future, would provide valuable insights into the evolution and potential control strategies for porcine rotavirus. Besides, it is important to note that the results of pig experiments may vary due to factors such as the virus strains, age of the pigs and viral challenge dosage. Nonetheless, PoRV infection consistently leads to clinical disease, microscopic lesions and viral shedding. Liu et al. (19) demonstrated PoRV could promote porcine epidemic diarrhea virus (PEDV) infection by targeting glutamine metabolism. Therefore, PoRV should be considered in the investigation of gastroenteritis outbreaks in the field, particularly in cases where PEDV is negative.

**Table 1:** The pathogenicity of different PoRVA strain isolated in China.

Year	Strain	Piglets	Dose	Clinical symptoms	References
2016	HJ G5P[7]	2-day-old	2 mL 10^7.6^ TCID_50_/mL	100% piglets developed obvious diarrhea at 12 HPI^a^ Two piglets died at 24 HPI. 100% mortality	In Chinese
2018	HN03 G9P[23]	3-day-old	2 mL 10^7.6^ TCID_50_/mL	100% piglets developed severe watery diarrhea within 24 HPI Piglets recovered at 72 HPI. No death	(16)
2022	SCJY-13 G9P[23]	2-day-old	2 mL/(no data)	Diarrhea started at 11 HPI and 100% diarrhea at 20 HPI 1 died at 30 HPI and 1 died at 44 HPI. 28.6% mortality	In Chinese
2022	CN127 G12P[7]	1-day-old	4 mL 10^7.6^ TCID_50_/mL	Diarrhea started at 6–24 HPI and persisted until euthanasia 3 piglets became lethargic from 60 HPI to 86 HPI and were euthanized	(18)
2022	AY01 G5P[23]	1-day-old	1 mL 10^6^ TCID_50_/mL	All 3 infected piglets occurred diarrhea and were euthanized at 48 HPI	In Chinese
2023	GX9579 G9P[23]	1-day-old	2 mL 10^7.46^ TCID_50_/mL	All 3 infected piglets occurred diarrhea 1 died at 30 HPI, 1 died at 44 HPI. 66.67% mortality	In Chinese
2023	JS G5P[23]	15-day-old	1 mL 10^6^ TCID_50_/mL	All 8 piglets exhibited diarrhea at 72 HPI 1 piglet died at 72 HPI and 2 at 144 HPI. 37.5% mortality	(17)
2024	AHFY2022 G9P[23]	5-day-old	2 mL 10^6.5^ TCID_50_/mL	4/5 piglets showed mild–severe diarrhea at 36 HPI 5/5 piglets occurred diarrhea at 48 HPI and persistent 1 day. No death	(12)
2024	AHFY2022 G9P[23]	27-day-old	2 mL 10^6.5^ TCID_50_/mL	1/5 piglets showed mild diarrhea after 24 HPI 5/5 piglets occurred diarrhea at 60 HPI and persistent 3 days. No death	(12)

a Hours post infection.

It has demonstrated that rotavirus can spread extra-intestinally and caused lung damage following PoRV infection in recent years (20). Nelsen et al. (21) identified a high frequency of porcine rotavirus A detection in lungs from conventional pigs with respiratory disease and virus genes were mainly localized in alveolar macrophages and bronchiolar epithelial cells *in situ* hybridization using RNAscope. The presence of viral RNA, VP6 antigen, and associated lesions in the lungs of pigs infected with AHBZ2303 and AHBZ2304 suggests that the lungs may serve as a target for rotavirus infection. Further studies were warranted to investigate PoRV prevalence using nasal swabs and lung samples, as well as to explore the potential mechanism underlying lung infection and the role of PoRV in respiratory disease.

Substantial evidence has indicated the important role for the commensal gut microbiota in enteric viruses’ infection and pathogenesis (22, 23). We tried to explore the changes of microbiota, and although significant changes were also determined upon AHBZ2303 or AHBZ2304 infection, the critical interactions between the host gut microbiota and PoRV infection need to be further studied. Besides, the increased production of IL-1, IL-6, IL-8, TNF-α and TGF-β in the jejunum were detected, indicating that AHBZ2303 or AHBZ2304 infection could induce host inflammatory response and contribute to inflammatory bowel disease, accompanied by significant pathological damage. So, addressing intestinal inflammation presents a vital strategy for mitigating diarrhea induced by porcine rotavirus.

## 5 Conclusion

In summary, the pathogenicity of AHBZ2303 (G11P[7]) and AHBZ2304 (G9P[23]) was analyzed, revealing fetal virulence for newborn colostrum-deprived piglets, characterized by obvious clinical signs, high virus shedding, microbiota changes and elevated inflammatory cytokines. Our study enriched the pathogenic data of porcine rotavirus and provided valuable reference materials for investigating the molecular mechanism underlying viral virulence.

## Data Availability

The data on gut microbiota presented in the study are deposited in the NCBI repository, accession number PRJNA1227677.
