# Supplementary material for: High pathogenicity of emerging porcine G9P[23] and G11P[7] rotavirus for newborn piglets in China
Source: Front Vet Sci. 2025 Apr 2;12:1531861. doi: 10.3389/fvets.2025.1531861 (PMC12031660; doi:10.3389/fvets.2025.1531861)
Supplement: Supplementary file 1 [file Table_1.DOCX]

**Supplementary Table 1. qRT-PCR primers used in the study.**

| **Gene** | **Primer** | **Primer sequence (5'-3')** |
| --- | --- | --- |
| Nsp5 | Forward | TGGTAGGAACGAACAGTATG |
|  | Reverse | TTGAAGCAGAATCAGATGGT |
| IL-1α | Forward | AAGCTGAGCCTCCAGAAGAAGG |
|  | Reverse | CAGGGCTCCCAGCTACTACCATA |
| IL-1β | Forward | CAATTCAGGGACCCTACCCTC |
|  | Reverse | ACGTTTCAAGGATGATGGGCT |
| IL-6 | Forward | GGCATCACCTTTGGCATCTTC |
|  | Reverse | CCTTCAGTCCAGTCGCCTTCT |
| IL-8 | Forward | CAGTTCTGGCAAGAGTAAGT |
|  | Reverse | CTCAATCACTCTCAGTTCCT |
| TNF-α | Forward | CGTTGTAGCCAATGTCAAAGCC |
|  | Reverse | TGCCCAGATTCAGCAAAGTCCA |
| TGF-β | Forward | AGCACAATGATCTGGCCGTT |
|  | Reverse | GCTGAAAGGTGTGACACGGA |
| β-actin | Forward | GGCATCCACGAAACTACCTT |
|  | Reverse | TGATCTCCTTCTGCATCCTG |
