# Supplementary material for: High pathogenicity of emerging porcine G9P[23] and G11P[7] rotavirus for newborn piglets in China
Source: Front Vet Sci. 2025 Apr 2;12:1531861. doi: 10.3389/fvets.2025.1531861 (PMC12031660; doi:10.3389/fvets.2025.1531861)
Supplement: Supplementary file 2 [file Presentation_1.PPTX]

## Slide 1
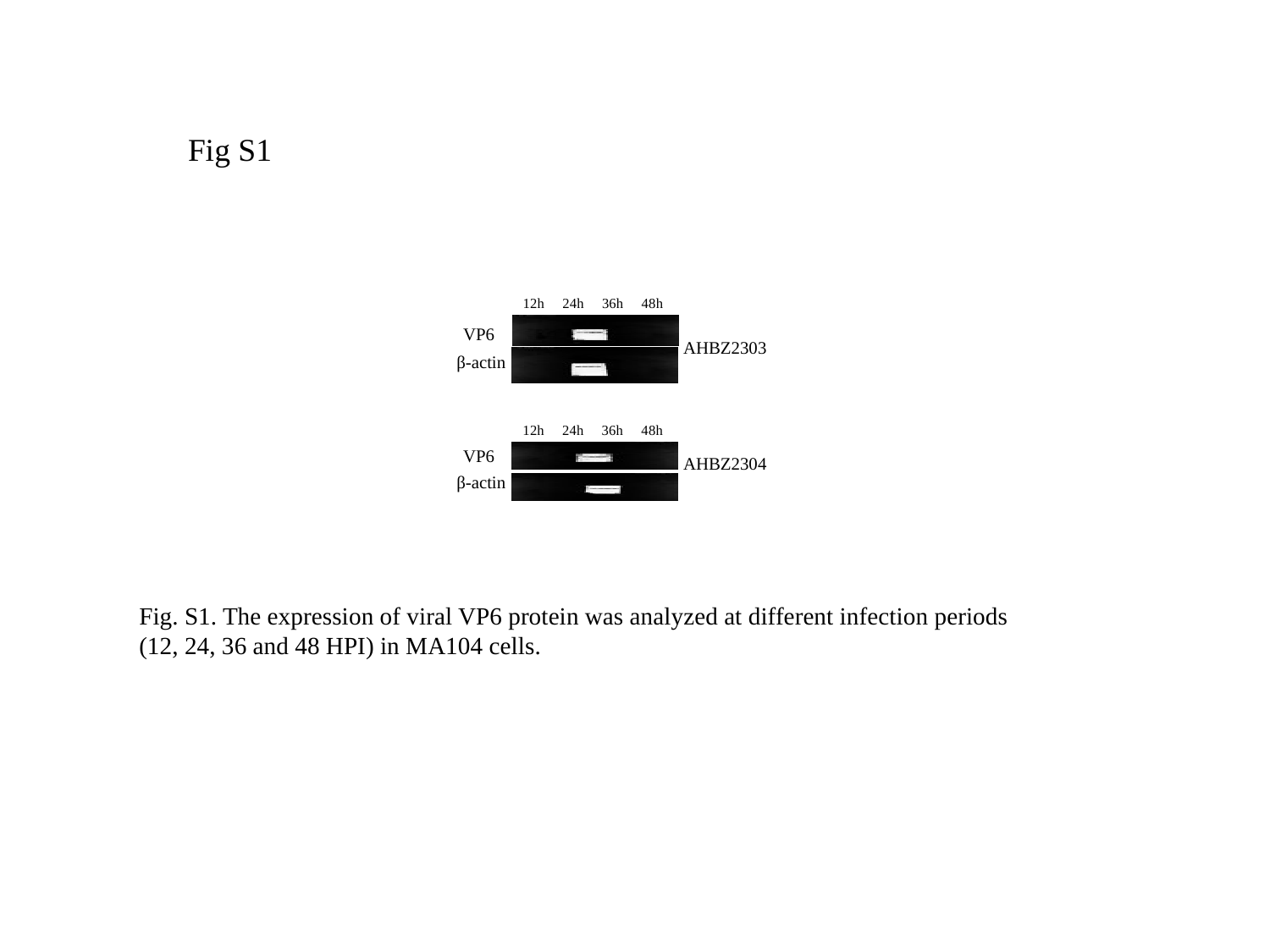

Fig S1
 12h 24h 36h 48h
VP6
AHBZ2303
β-actin
 12h 24h 36h 48h
VP6
AHBZ2304
β-actin
Fig. S1. The expression of viral VP6 protein was analyzed at different infection periods (12, 24, 36 and 48 HPI) in MA104 cells.
